# Supplementary material for: Few-fermion resonant tunneling and underbarrier trapping in asymmetric potentials
Source: Commun Phys. 2025 Jun 20;8(1):259. doi: 10.1038/s42005-025-02189-9 (PMC12181076; doi:10.1038/s42005-025-02189-9)
Supplement: Supplementary file 1 — Supplemental Material [file 42005_2025_2189_MOESM1_ESM.pdf]

nature portfolio

Peer Review File

Few-fermion resonant tunneling and underbarrier trapping in asymmetric potentials

Corresponding Author: Professor Denys Bondar

This file contains all reviewer reports in order by version, followed by all author rebuttals in order by version.

Version 0:

Reviewer comments:

Reviewer #1

(Remarks to the Author)
In this work, the authors explore the emergent tunneling dynamics of a few spin fermion systems trapped in discrete asymmetric few lattice sites. It is first numerically shown that in the noninteracting limit the fermions undergo the same tunneling behavior if placed at different sites of the barrier. This mechanism is also proved in a corresponding appendix. As a next step, the impact of interactions on the tunneling dynamics of the fermions is analyzed. In fact, it is demonstrated that interactions break the previous tunneling symmetry resulting in non-trivial asymmetric tunneling behavior. Insights from the Falicov-Kimball limit according to which the observed dynamics can be interpreted due to an effective interaction-induced shift in the barrier height are provided. The interplay of the initial spin configurations, the interactions and the barrier asymmetry is also discussed for somewhat larger spin systems. For instance, it is argued that spin-triplet states lead to asymmetric response. In addition, depending on the interactions, the phenomena of under-barrier resonant trapping and many-body resonant tunneling are revealed.

I find the results interesting and that they may possible inspire related future applications in ultracold atom platforms or nanoscale devices. The considered setting can be indeed readily realized using ultracold atoms according to relevant experimental demonstrations. The method used appears to be solid. However, I am not convinced about the suitability of the work to Communications Physics since to my opinion the current version neither conveys the significant improvement of our understanding on few-body tunneling mechanisms or related properties nor justifies the broader impact of the present results. In addition, I have various comments and suggestions not only regarding the discussion of the results and their presentation in the paper but also for further analysis. For these reasons, I am not yet convinced about the suitability of the manuscript for Communications Physics. If the authors are able to address the points listed below and accordingly substantially revise their manuscript by providing more detailed explanations and further analysis, I believe that the work can be reconsidered. Specific recommendations follow

1) In the third to the last sentence of the abstract in the statement "…and fundamentally…" something is missing e.g. it should read "…and being fundamentally…".

2) In the second paragraph of the left column of the introduction, it is stated that to better understand the role of correlations an approach is to study few-body systems which capture the essential properties and provide insights into the major phenomena of the systems under investigation. It would be nice to be more specific at this point and, for instance, mention that few-body systems allow to assess also the microscopic mechanisms of the studied dynamical processes.

3) In the second paragraph of the right column in the introduction, I would suggest to add a few more references about the dynamics of few-body systems especially for fermions and not only references 12-16 some of which in fact refer to bosons. Examples of such references could be, for instance, Scientific Reports 12, 19710 (2022) and New Journal of Physics 22, 063058 (2020) as well as others on this topic.

4) I suggest the authors to emphasize more the novelty of their results in the introduction and provide whenever possible brief statements about phenomena that have been explored before. As an example, currently, the penultimate paragraph of the introduction describing the results does not make clear which ones are reported here for the first time or which ones advance our understanding compared to previous studies.

5) I find the argument in the last sentence of the introduction stating that the results on the asymmetric tunneling can be applied to design nanoscale devices with tailored transport properties, such as transistors, memristors, and sensors too broad. I would suggest to provide a corresponding (even brief) justification.

6) In Section II, when introducing the spin Hamiltonian it would be good to clarify that it refers to a one-dimensional situation. Here, it would also worth mentioning again that this can be experimentally realized and refer to some of the experiments provided in the introduction. Along the same lines, I suggest to mention that the tunneling and on-site interaction terms can be tuned in the experiment.

7) I suggest to add a brief discussion on the computational method used, i.e. the open-source QuSpin package, such that the non-expert reader can be able to understand the main aspects of this method and the manuscript is self-contained. Currently, it is just mentioned that this method is used and two relevant references are provided. Here, also the numerical convergence of the results should be discussed.

8) I suggest to explicitly provide the functional form of the external potential. Currently, it is only schematically shown in Figure 1.

9) In Figure 1, it does not become clear what the gray circles represent. Do they indicate the sites of the lattice? If yes please clarify in the caption. Also, why the solid black lines connect only three of them. A further clarification is needed here.

10) In the first paragraph of Section II B, I believe that there is a typo in the sentence "…agree overlap perfectly". Please rectify this issue.

11) In the captions of Figures 2, 4, 6 and 7, it is stated that the time-evolution is normalized to the hopping amplitude. I suggest to show this explicitly in the corresponding axis instead of quoting just "t" which I guess is the simulation time. Indeed, these timescales would facilitate the realization of the system under consideration by a corresponding experiment. To my opinion, it would also worth to add a statement in the manuscript whether such timescales can be tracked by current experiments and provide a few relevant references.

12) Along the same lines, an arising question for all the cases discussed is what happens with the tunneling for longer evolution times. Is the overall tunneling amplitude and frequency constant or do they exhibit any dephasing? From the current timescales it seems that the amplitude is not constant but it is difficult to have any conclusion about the frequencies. If this is the case how can one interpret corresponding modifications?

13) In Figure 2(a), it can be seen that the expectation value of the number operator features high-frequency amplitude oscillations. Where do the underlying frequencies correspond to? Is it possible to be identified in terms of eigenstate transitions?

14) The fermion arrangement and the effective change of the barrier for one frozen particle presented in Figure 3 should be explained in the caption. In fact, the case of panel (b) is not even explained in the main text but rather it is mentioned that "… whereas the second case [see Fig. 3(right)]…". Please elaborate further.

15) At the end of the first paragraph of the right column of page 3, it is stated that "Thus the asymmetry in n_L is caused by penetration through two different barriers…". This is not clear especially since just one sentence before it is claimed that "inter-particle interaction U can be treated as an effective change in the external potential". This discussion certainly needs further clarification.

16) Is the tunneling dynamics shown in Figure 4 independent of the interaction strength U? If yes, please explain why this is the case. If, however, this is not true I would suggest to add a heat map showing the time-evolution of the number operator with respect to the interaction strength U. Is it possible to enter more complex or simpler tunneling channels? At which interactions tunneling is prohibited? Please elaborate.

17) In the caption of Figure 6, there is a statement "The circle in the schematic barrier illustration…". To which circle and at which barrier illustration do the authors refer to here? Please clarify.

18) Upon increasing the system size it is claimed that the phenomenon of "Underbarrier resonant trapping" is observed. However, this mechanism is not explained in depth to my opinion. Is there any corresponding transition of spin states that characterizes this behavior? Is this a finite size effect? Under which circumstances can it be present in larger systems? I find the discussion around this interesting mechanism currently suppressed.

19) In general, to my point of view, it would worth to attempt to explain the observed tunneling mechanisms in terms of transitions in the underlying eigenspectrum if possible.

20) It does not become clear whether two-body contributions are participating in the discussed tunneling channels. Is it possible to calculate corresponding two-body probabilities instead of only the number operator? If two-body contributions are negligible it should be explicitly mentioned.

21) In all Figure captions I suggest to add a one sentence description of the main message conveyed at the beginning.

22) The Appendix may be moved to a supplemental material where more details about the construction of the path integral can be provided, e.g. the action that is employed. Also, some relevant references (even if this means textbooks) would be nice to be provided.

Reviewer #2

(Remarks to the Author)
Having read the abstract of the manuscript I've thought that this paper reports new results on the scattering problem for interacting particles, in spirit of previous studies of this problem [see, for example, A.R.Kolovsky, J.Link, and S.Wimberger, Energetically constrained co-tunneling of cold atoms, New J. of Phys. 14, 075002 (2012)]. However, it appeared that the authors addressed a much simpler problem of quasi-periodic dynamics of a finite number of fermions in a finite tight-binding lattice with on-site energies corresponding to a potential barrier in the lattice centrum. As the initial condition the authors consider the situation where at \$t=0\$ all fermions reside in front of the barrier and, thus, the probability to find a fermion behind the barrier is strictly zero. For \$t>0\$ this probability is generally not zero and shows quasi-periodic oscillation. Then, the average of this quasi-periodic process over time is termed tunneling probability.

I am pretty sure that the presented numerical results are correct, however, I am not sure that the calculated quantity can be referred to as the tunneling probability of interacting fermions. Indeed:

(i) One of the authors conclusions is that the tunneling probability of the bound pair of fermions (doublon) is asymmetric. This implies, in particular, that doublons of cold atoms loaded into the sawtooth optical lattice should exhibit directed current. This would be a wonderful effect but I have never heard of it and I am afraid that it does not exist;

(ii) Presented numerical results clearly indicate a possibility for doublon dissociation. Thus, one should distinguish between tunneling and dissociation probabilities;

(iii) time-averaged quantities may depend on the interval \$T\$. There is no comment on this issue in the manuscript.

To summarize, I consider this work as preliminary studies before addressing the more experimentally relevant problem of the doublon tunneling dynamics in a sawtooth potential. For this reason I, to my great regret, cannot recommend this manuscript for publication in Communications Physics.

Reviewer #3

(Remarks to the Author)
I advice for publication after a major revision. Please find my detailed comments and recommendation in the attached pdf file.

Version 1:

Reviewer comments:

Reviewer #1

(Remarks to the Author)
The authors have addressed satisfactorily most of the questions and concerns raised in my previous report and accordingly amended their revised manuscript. In this sense, the presentation is improved by means that the origin of the different tunneling mechanisms involved (e.g. underbarrier resonant trapping and many-body resonant tunneling) is clarified and better explained. Also, the Introduction is improved, stating more clearly the motivation of this work which is now better embedded in the current literature and the new results are better conveyed. In addition, the potential applications of the results have been clarified and the figure captions have been improved easing the understanding of the reader. Moreover, the numerical computations have been checked carefully and the distinction between spinor and pseudo-spinor states along with the spin symmetries of the setup have been discussed in more detail. Additionally, as I mentioned in my original report the topic of this work is indeed interesting. A few final suggestions that I have, before acceptance are the following:

1) Explicitly mention why and how the single-particle tunneling processes, such as the many-body resonant tunneling, discussed in the main text are different from the previously known single-particle resonant tunneling. I think this distinction should be made clear in order to ease the understanding of the reader, especially the non-expert one.

2) A natural question that emerges is whether any of the underbarrier resonant trapping or many-body resonant tunneling have classical analogues. Can the authors comment on this issue?

3) Mention the degree of convergence of the time-averaged probabilities (number operator) with respect to the considered evolution time. Currently, the statement that the relevant observables are largely unaffected by increasing the final time T is a bit general.

4) It would be nice to see a scaling of the time required to reach site j* with respect to the system size for the underbarrier resonant trapping. This will assist the experimental realization of this phenomenon.

5) Please check carefully the entire manuscript for typographical errors.

Otherwise, I support publication of this work in Communications Physics.

Reviewer #2

(Remarks to the Author)
The authors invested considerable time in answering the reviewers' questions and essentially improved the manuscript. (In fact, some of these questions have already been addressed in the paper mentioned in my previous report which the author forgot to cite in the amended manuscript.) In its present form I would classify this work as an analysis of the toy model which shows the asymmetry of the dublon dissociation due to the underbarrier resonant trapping of one of the two particles. To bring this toy model closer to laboratory experiments with cold atoms (to which the authors constantly refer to) one should consider larger lattices, see remark No.18 of the first Reviewer. In this respect I would recommend substituting the right panel in Fig.5 in the manuscript for the right panel in Fig.5 in the response letter, where one can see how the dublon hits the barrier. And, of course, the work by Kolovsky, et al. of 2012 has to be cited because it discusses the same phenomenon of the resonant trapping but for the pair of interacting bosons. (Please, also check the recent paper "Two-particle tunneling and the impact of interaction" by Brugger, Dittel, and Buchleitner published in Physical Review A.) Provided these remarks are taken into account the work can be published in Communications Physics.

Reviewer #3

(Remarks to the Author)
The referees have addressed my comments in a satisfactory manner in their revised manuscript and
I would support its publication in Communications Physics. In the attached pdf there are some minor comments that the authors might find useful for a final polish of their manuscript before publication.

Version 2:

Reviewer comments:

Reviewer #1

(Remarks to the Author)
The revised manuscript addresses my final comments raised in the second report. In particular, a discussion regarding the distinction of the many-body resonant tunneling with the known single-particle resonant tunneling process has been added and a scaling of the time required to reach site j* with respect to the system size has been incorporated as a supplementary note. Moreover, Figure 5 has been updated containing the density dynamics for L=20 sites. Finally, the degree of convergence of the number operator has been clarified. For these reasons, I recommend publication of the manuscript in Communication Physics in its current form.

Open Access This Peer Review File is licensed under a Creative Commons Attribution 4.0 International License, which permits use, sharing, adaptation, distribution and reproduction in any medium or format, as long as you give appropriate credit to the original author(s) and the source, provide a link to the Creative Commons license, and indicate if changes were made.
In cases where reviewers are anonymous, credit should be given to 'Anonymous Referee' and the source.
The images or other third party material in this Peer Review File are included in the article's Creative Commons license, unless indicated otherwise in a credit line to the material. If material is not included in the article's Creative Commons license and your intended use is not permitted by statutory regulation or exceeds the permitted use, you will need to obtain permission directly from the copyright holder.
To view a copy of this license, visit https://creativecommons.org/licenses/by/4.0/

TITLE: Few-fermion resonant tunneling and underbarrier trapping in asymmetric potentials
AUTHORS: Elvira Bilokon, Valeriia Bilokon, Dusty R. Lindberg, Andrii Sotnikov, Lev Kaplan, and Denys I. Bondar

We sincerely appreciate the time and effort the Referees have dedicated to reviewing our manuscript. Their insightful comments and suggestions have been valuable in improving the clarity, accuracy, and presentation of our work.

We have carefully revised the manuscript to address the concerns raised. Below, we provide a detailed point-by-point response to each comment, outlining the modifications we have made. Modified (or new) parts of the text have been marked in green in the PDF file.

Summary of main modifications:

1. **Abstract:**
   - We have corrected a grammatical issue in the abstract for improved readability. (R1.1)
2. **Introduction:**
   - We have added a sentence to explicitly highlight that few-body systems allow for the investigation of microscopic mechanisms, including tunneling dynamics, correlation effects, and quantum entanglement. (R1.2)
   - We have expanded the list of references on few-body dynamics, especially for fermionic systems, incorporating suggested papers and additional relevant studies. (R1.3)
   - We have emphasized the novelty of the results by distinguishing new findings from previously known phenomena. (R1.4)
   - We have briefly outlined the potential application of asymmetric tunneling in nanoscale devices. (R1.5)
   - We have expanded the introduction to emphasize the broader relevance of tunneling control beyond ultracold atoms. (R3.2)
3. **Model:**
   - We have explicitly stated that the spin Hamiltonian refers to a one-dimensional system and clarified that tunneling and on-site interactions can be experimentally tuned. (R1.6)
   - We have explicitly provided the functional form of the external potential in the main text. (R1.8)
   - We have clarified the Figure 1 caption, explaining the representation of lattice sites and the purpose of solid black lines. (R1.9)
4. **Results:**
   - We have fixed a typo. (R1.10; R3.5)
   - We have added a brief explanation of the derivation of the theorem. (R3.4)
   - We have adjusted figure captions (Figures 2, 4, 6, and 7) to explicitly indicate time normalization to the hopping amplitude. (R1.11)
   - We have mentioned that current cold-atom experiments can track the discussed timescales and provided supporting references. (R1.11)
   - We have added additional comments in the manuscript regarding the behavior of larger systems to provide further clarification. (R1.18)
   - We have explicitly stated that the manuscript does not claim that the doublon tunnels as a whole. (R1.20; R2.1)

- We have clarified that the primary contribution to $\langle \hat{n}_L \rangle$ originates from a single particle after doublon dissociation. (R2.2)
- We have updated captions for all the figures. (R1.21)
- We have added a comment in the manuscript to highlight the robustness of the findings with respect to T. (R2.3)
- We have revised the discussion in the *Results* section to reflect that singlet states exhibit asymmetric tunneling, while triplet states preserve symmetry. (R3.1)
- Added a brief discussion on $\hat{\mathbf{S}}^2$ symmetry in the *Model* subsection. (R3.1)
- We have explained the choice of a six-site system in *Symmetry considerations.* (R3.1)

5. **Methods:**

- We have expanded the description of the computational method (QuSpin package). (R1.7)

6. **Supplementary Material:**

- We have moved an extended discussion on the Falicov-Kimball limit to the *Supplementary Note 1* (R1.14-15; R3.3).
- We have moved the Appendix to the *Supplementary Note 2.* (R1.22)

Responses to the Referee comments

Referee 1

1

In the third to the last sentence of the abstract in the statement "...and fundamentally..." something is missing e.g. it should read "...and being fundamentally...".

We are thankful to the Referee and have revised the sentence.

2

In the second paragraph of the left column of the introduction, it is stated that to better understand the role of correlations an approach is to study few-body systems which capture the essential properties and provide insights into the major phenomena of the systems under investigation. It would be nice to be more specific at this point and, for instance, mention that few-body systems allow to assess also the microscopic mechanisms of the studied dynamical processes.

We have rewritten the relevant section of the introduction to explicitly mention the study of microscopic mechanisms (see page 3 of the revised manuscript).

3

In the second paragraph of the right column in the introduction, I would suggest to add a few more references about the dynamics of few-body systems especially for fermions and not only references 12-16 some of which in fact refer to bosons. Examples of such references could be, for instance, Scientific Reports 12, 19710 (2022) and New Journal of Physics 22, 063058 (2020) as well as others on this topic.

We have expanded the list of references in the relevant section of the introduction to include the suggested papers (Scientific Reports 12, 19710 (2022) and New Journal of Physics 22, 063058 (2020)), along with a few additional references that further contribute to the discussion of fermionic few-body dynamics.

4

I suggest the authors to emphasize more the novelty of their results in the introduction and provide whenever possible brief statements about phenomena that have been explored before. As an example, currently, the penultimate paragraph of the introduction describing the results does not make clear which ones are reported here for the first time or which ones advance our understanding compared to previous studies.

We thank the Referee for this valuable suggestion. To address this, we have revised the corresponding paragraph to better highlight the novelty of our findings and distinguish them from previously studied phenomena (see page 3 of the modified manuscript).

5

I find the argument in the last sentence of the introduction stating that the results on the asymmetric tunneling can be applied to design nanoscale devices with tailored transport properties, such as transistors, memristors, and sensors too broad. I would suggest to provide a corresponding (even brief) justification.

We appreciate the Referee's suggestion. We now briefly outline how interaction-induced asymmetry in quantum transport could be leveraged in nanoscale device design (see page 4 of the modified manuscript).

6
In Section II, when introducing the spin Hamiltonian it would be good to clarify that it refers to a one-dimensional situation. Here, it would also worth mentioning again that this can be experimentally realized and refer to some of the experiments provided in the introduction. Along the same lines, I suggest to mention that the tunneling and on-site interaction terms can be tuned in the experiment.

We have updated this section (see section *Results*, subsection *Model*) to explicitly state that the spin Hamiltonian refers to a one-dimensional system. Additionally, we have included references to relevant experiments mentioned in the introduction that demonstrate the experimental realization of such systems. Furthermore, we have mentioned that both the tunneling and on-site interaction terms can be tuned in experiments.

7
I suggest to add a brief discussion on the computational method used, i.e. the open-source QuSpin package, such that the non-expert reader can be able to understand the main aspects of this method and the manuscript is self-contained. Currently, it is just mentioned that this method is used and two relevant references are provided. Here, also the numerical convergence of the results should be discussed.

In the section *Methods* we have provided an additional information regarding QuSpin Package. Regarding numerical convergence, the exact diagonalization (ED) method provides numerically exact results, as it solves the full Hamiltonian matrix without approximation. For the time evolution, the expansion order of $e^{-i\hat{\mathcal{H}}t}$ is not manually controlled. Instead, the QuSpin package automatically determines the number of terms in the Taylor series expansion to ensure accuracy.

To demonstrate the accuracy of our results, the initial wavefunction can be expanded in the eigenstate basis. The time evolution is then given by

$$|\Psi(t)\rangle = \sum_n e^{-iE_nt}|n\rangle\langle n|\Psi(0)\rangle, \tag{1}$$

where the sum runs over all eigenstates of the Hamiltonian, with E_n denoting the energy corresponding to the eigenvector $|n\rangle$.

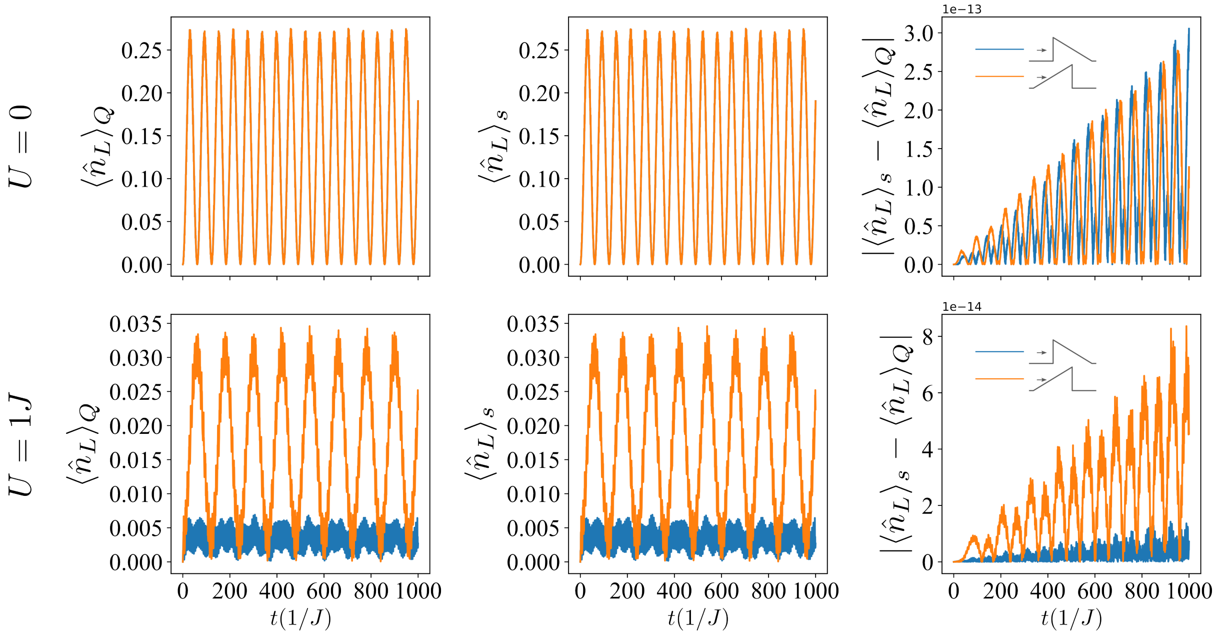

Figure 1: Left: time evolution of $\langle\hat{n}_L\rangle$ calculated by function from QuSpin package. Middle: time evolution of $\langle\hat{n}_L\rangle$ calculated by the direct exponentiation. Right: the absolute value of the difference between two results.

For the system size $L = 4$, Fig. 1 shows the dependence of $\langle \hat{n}_L \rangle$ on time calculated in two different ways. The right column shows the result obtained by the *obs_vs_time* function from QuSpin package ($\langle \hat{n}_L \rangle_Q$), while the middle column represents the time evolution of $\langle \hat{n}_L \rangle_s$ calculated by the direct exponentiation. As one can see from the very right column, over relatively large timescale, the difference between $\langle \hat{n}_L \rangle_Q$ and $\langle \hat{n}_L \rangle_s$ is of machine precision order.

8

I suggest to explicitly provide the functional form of the external potential. Currently, it is only schematically shown in Figure 1.

We appreciate the Referee's suggestion to explicitly provide the functional form of the external potential. To address this, we have added the mathematical expression of the external potential in the main text (see Eq. 2 in the section *Results*, subsection *Model*).

9

In Figure 1, it does not become clear what the gray circles represent. Do they indicate the sites of the lattice? If yes please clarify in the caption. Also, why the solid black lines connect only three of them. A further clarification is needed here.

We have updated the caption of Fig. 1 in the main text (on page 6 of the revised version) to explicitly state that the gray circles represent the sites of the lattice. Additionally, we have clarified that the solid black lines are not intended to connect lattice sites but rather to illustrate the triangular shape of the barrier.

10

In the first paragraph of Section II B, I believe that there is a typo in the sentence "...agree overlap perfectly". Please rectify this issue.

We appreciate the Referee for noticing this typo. We have corrected the sentence.

11

In the captions of Figures 2, 4, 6 and 7, it is stated that the time-evolution is normalized to the hopping amplitude. I suggest to show this explicitly in the corresponding axis instead of quoting just "t" which I guess is the simulation time. Indeed, these timescales would facilitate the realization of the system under consideration by a corresponding experiment. To my opinion, it would also worth to add a statement in the manuscript whether such timescales can be tracked by current experiments and provide a few relevant references.

We have mentioned that modern cold-atom experiments can reach comparable timescales, and we have added references to relevant experimental studies (see page 6). Additionaly, we have modified the captions of Figs. 2, 4, 6, and 7 following the suggestion.

12

Along the same lines, an arising question for all the cases discussed is what happens with the tunneling for longer evolution times. Is the overall tunneling amplitude and frequency constant or do they exhibit any dephasing? From the current timescales it seems that the amplitude is not constant but it is difficult to have any conclusion about the frequencies. If this is the case how can one interpret corresponding modifications?

We have plotted the time evolution of $\langle \hat{n}_{\text{after}} \rangle$ for longer evolution times. As one can see from Fig. 2, in the noninteracting case the overall tunneling amplitude as well as frequency are constant over the entire timescale.

With the presence of interaction in the system, the dynamics becomes more complicated. Based on our simulations (see Fig. 3), one can conclude that, in general, the overall amplitude and frequency are not constant.

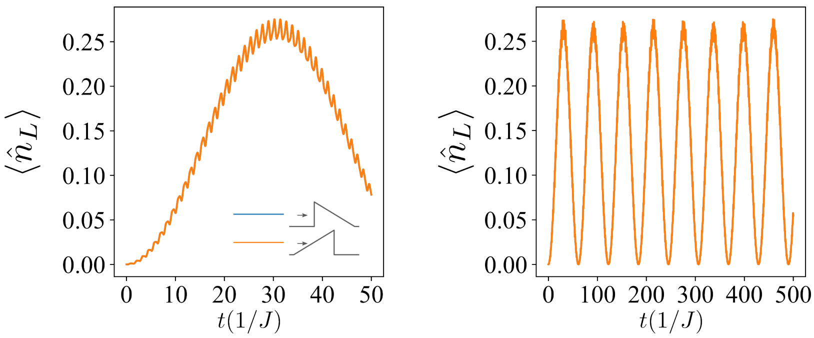

Figure 2: For the $L = 4$ system with a noninteracting ($U = 0$) doublon as an initial state, the expectation value of $\langle \hat{n}_L \rangle$ as a function of time for (left): $t \in [0, 50]$; (right): $t \in [0, 500]$. The height of the barrier is $h = 10J$.

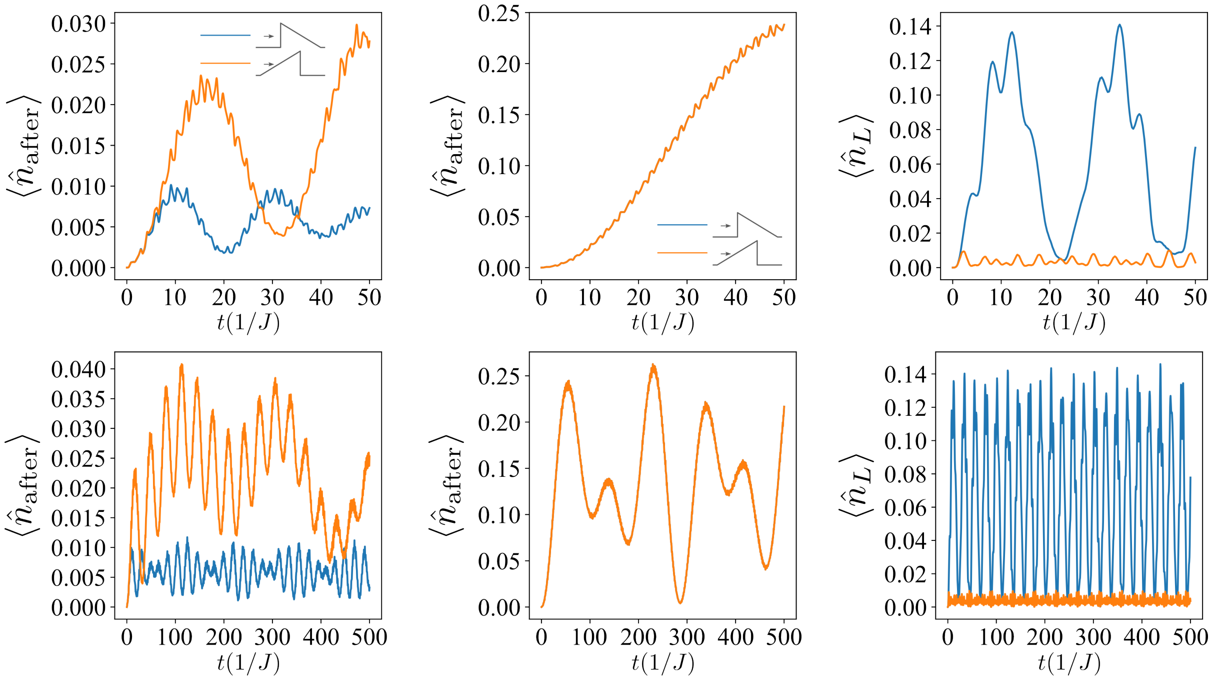

Figure 3: For the $L = 6$ system with interacting ($U = 0.5J$) (right): $|\Psi(0)\rangle = \frac{1}{\sqrt{2}}(| \uparrow_1\downarrow_2\rangle - | \downarrow_1\uparrow_2\rangle)$ and (middle): $|\Psi(0)\rangle = \frac{1}{\sqrt{2}}(| \uparrow_1\downarrow_2\rangle + | \downarrow_1\uparrow_2\rangle)$ as an initial state, the expectation value of $\langle \hat{n}_{\text{after}} \rangle$ as a function of time. (Left): for the $L = 4$ system with interacting ($U = 10J$) doublon as an initial state, the expectation value of $\langle \hat{n}_L \rangle$ as a function of time. (Up): $t \in [0, 50]$; (down): $t \in [0, 500]$. The height of the barrier is $h = 10J$ for $L = 6$, and $h = 20J$ for $L = 4$.

13

In Figure 2(a), it can be seen that the expectation value of the number operator features high-frequency amplitude oscillations. Where do the underlying frequencies correspond to? Is it possible to be identified in terms of eigenstate transitions?

To investigate this question, we performed the Fast Fourier Transform (FFT) of $\langle \hat{n}_L \rangle$ for a non-interacting system with $L = 4$ sites. As shown in Fig. 4(a), there are two prominent peaks at $\Delta E_1 \approx 5J$ and $\Delta E_2 \approx 10J$. However, it is clear from Fig. 4(b) and Fig. 4(c) that these energy differences correspond to multiple pairs of eigenvalues. This suggests that the oscillation frequencies arise from a superposition of many eigenstate transitions, making it difficult to attribute them to specific transitions in the eigenspectrum.

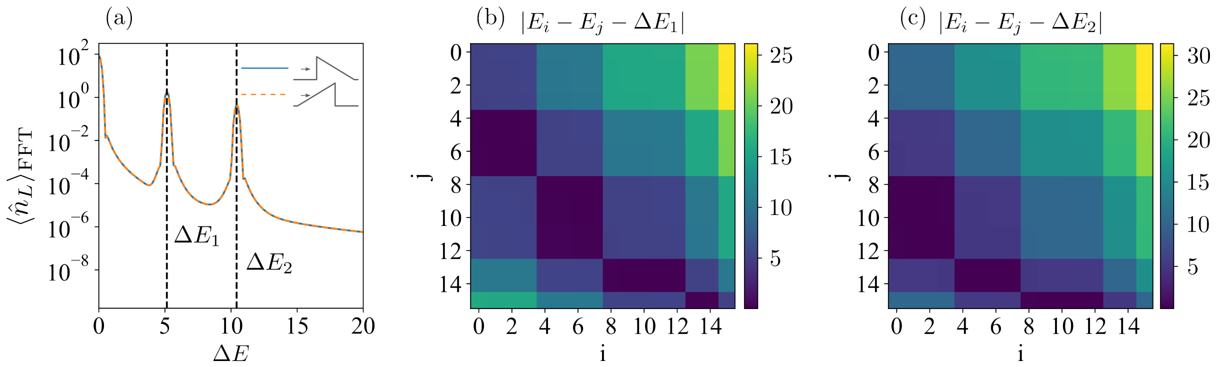

Figure 4: For $L = 4$ system size, (a) FFT of $\langle \hat{n}_L \rangle$ with $U = 0$ and external potential $h = 10J$. ΔE_1 (ΔE_2) represents the position of the first (second) peak. Heatmap of (b) $|E_i - E_j - \Delta E_1|$ and (c) $|E_i - E_j - \Delta E_2|$ where E_i and E_j are eigenvalues. The indices i and j represent eigenvalue numbers. The color scale indicates the magnitude of the deviation.

14

The fermion arrangement and the effective change of the barrier for one frozen particle presented in Figure 3 should be explained in the caption. In fact, the case of panel (b) is not even explained in the main text but rather it is mentioned that "...whereas the second case [see Fig. 3(right)]...". Please elaborate further.

We have revised the figure caption to explicitly describe the fermion configuration and have mentioned that the presence of a frozen particle modifies the barrier. Furthermore, we have expanded the discussion to provide a more detailed explanation of the scenario depicted in panel (b) (this section is moved to Supplementary materials in accordance with the recommendation of Referee 3, see *Supplementary Note 1, Fig. 1*).

15

At the end of the first paragraph of the right column of page 3, it is stated that "Thus the asymmetry in n_L is caused by penetration through two different barriers...". This is not clear especially since just one sentence before it is claimed that "inter-particle interaction U can be treated as an effective change in the external potential". This discussion certainly needs further clarification.

We have provided some explanation in the *Supplementary Note 1*. Our statement that "the inter-particle interaction U can be treated as an effective change in the external potential" refers to the fact that the presence of a spin-up particle modifies the local energy of a site, thereby effectively changing the external potential. However, as shown in Fig. 1 in *Supplementary Note 1*, the effective barriers that the spin-↑ particle encounters while tunneling from vertical or angled sides of the barrier are not mirror images of each other. This asymmetry leads to the asymmetry in $\langle \hat{n}_L \rangle$.

16

Is the tunneling dynamics shown in Figure 4 independent of the interaction strength U? If yes, please explain why this is the case. If, however, this is not true I would suggest to add a heat map showing the time-evolution of the number operator with respect to the interaction strength U. Is it possible to enter more complex or simpler tunneling channels? At which interactions tunneling is prohibited? Please elaborate.

The dependence of the tunneling dynamics on the interaction strength U depends on the choice of the initial state. If the initial state of the system is defined as $|\Psi(0)\rangle = \frac{1}{\sqrt{2}}(|\uparrow_1\downarrow_2\rangle - |\downarrow_1\uparrow_2\rangle)$ the tunneling dynamics does depend on the interaction amplitude U. As discussed in subsection *Symmetry considerations*, the action of the time-evolution operator $e^{-i\hat{\mathcal{H}}t}$ on this initial state generates terms that include interaction. However, if the system starts to evolve from state $|\Psi(0)\rangle = \frac{1}{\sqrt{2}}(|\uparrow_1\downarrow_2\rangle + |\downarrow_1\uparrow_2\rangle)$,

the dynamics is independent of U. Following the same procedure by acting with $e^{-i\hat{\mathcal{H}}t}$ on $|\Psi(0)\rangle$, the obtained state $|\Psi(t)\rangle$ at time t does not contain terms with the interaction amplitude U.

The way we defined our system, tunneling is allowed for all interaction strengths. However, for some values of U, it might be suppressed due to total energy conservation.

17

In the caption of Figure 6, there is a statement "The circle in the schematic barrier illustration. . . ". To which circle and at which barrier illustration do the authors refer to here? Please clarify.

To clarify that, we have revised the caption of the corresponding Figure. (Note that now it is Fig. 5 in subsection *Nontrivial dynamics in asymmetric potentials*). Namely, now one can read that *"The small schematic illustrations above each panel represent the potential barrier with circles indicating the site j^* where the potential is $h/2 = U$."*

18

Upon increasing the system size it is claimed that the phenomenon of "Underbarrier resonant trapping" is observed. However, this mechanism is not explained in depth to my opinion. Is there any corresponding transition of spin states that characterizes this behavior? Is this a finite size effect? Under which circumstances can it be present in larger systems? I find the discussion around this interesting mechanism currently suppressed.

To demostrate that the phenomenon is also observed in larger systems, we provide the Referee with Fig. 5, which illustrates the evolution of $\langle \hat{n}_j \rangle$ for $L = 16$ and $L = 20$ system sizes. While for $L = 4$ (see Fig. 5 of the revised manuscript) the effect of the underbarrier resonant trapping becomes apparent in a short period of simulation time, it takes longer to notice it for larger systems due to the increased number of evolution steps required to reach the j^* site.

As it was mentioned in the manuscript, the underbarrier resonant trapping results from the conservation of total energy. Hence, as long as the system evolves under this energy constraint, the effect can still be observed. The phenomenon is not a finite size effect, although it is noticeable more quickly in a smaller systems due to fewer available states.

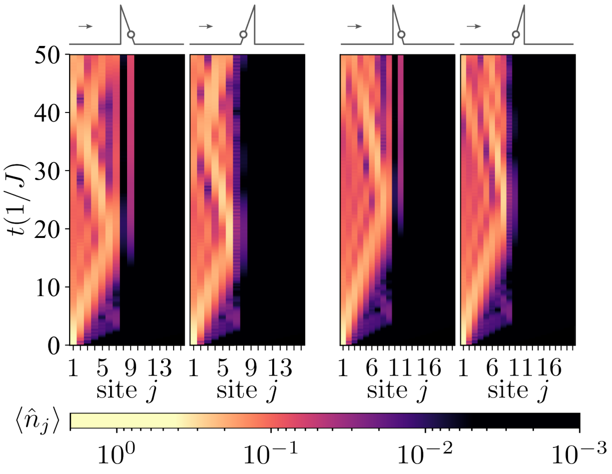

Figure 5: For different system sizes, $L = 16$ (left) and $L = 20$ (right), the evolution of $\langle \hat{n}_j \rangle$ is shown color-coded on a logarithmic scale. The initial state is a doublon positioned at the first site. The interaction strength is $U = 10J$. The small schematic illustrations above each panel represent the potential barrier with circles indicating the site j^* where the potential is $h/2 = U$.

We have added additional comments in the manuscript regarding the behavior of larger systems to provide further clarification on this point (see page 11 of the modified manuscript).

19
In general, to my point of view, it would worth to attempt to explain the observed tunneling mechanisms in terms of transitions in the underlying eigenspectrum if possible.

To explore this aspect, we performed the FFT of the $\langle \hat{n}_L \rangle$ with the presence of the interaction U (see Fig. 6). As one can see, the structure of the spectrum makes it challenging to directly associate specific peaks with dominant transitions between eigenstates. Due to this spectral complexity, we believe that an interpretation in terms of eigenstate transitions may not provide a clear or intuitive understanding of the tunneling dynamics.

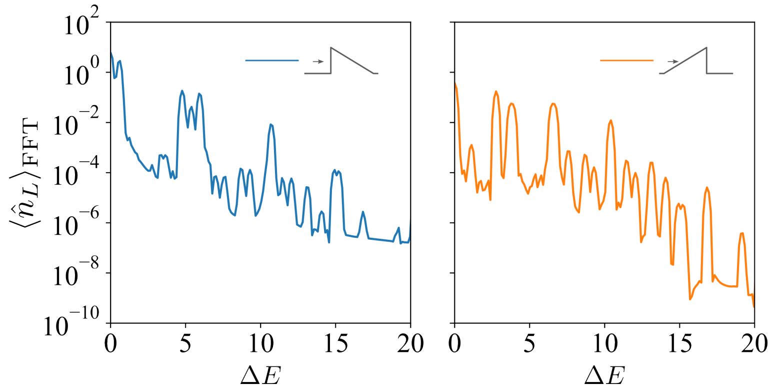

Figure 6: For $L = 4$ system size, FFT of $\langle \hat{n}_L \rangle$ with the presence of the interaction $U = 5J$ and external potential $h = 10J$. The initial configuration: a doublon is positioned before the vertical side (left) or the angled side (right) of the barrier.

20
It does not become clear whether two-body contributions are participating in the discussed tunneling channels. Is it possible to calculate corresponding two-body probabilities instead of only the number operator? If two-body contributions are negligible it should be explicitly mentioned.

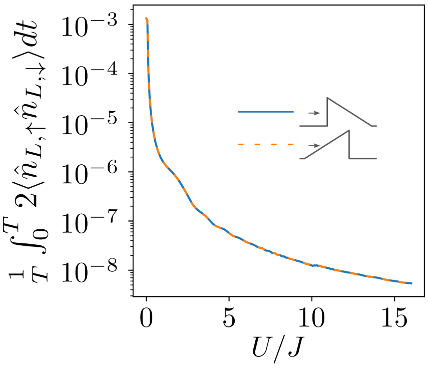

Figure 7: For the $L = 4$ system, the time-averaged $[T = 100/J]$ expectation value of $2\langle \hat{n}_{L,\uparrow}\hat{n}_{L,\downarrow}\rangle$ as a function of the interaction amplitude U. The initial state is a doublon positioned at the first site. The barrier height is $h = 20J$.

While the precise case the Referee referring to is unclear, to assess two-body contributions, we have calculated the time average of $2\langle \hat{n}_{L,\uparrow}\hat{n}_{L,\downarrow}\rangle$, see Fig. 7. It is clear that the two-body contributions are negligibly small. We also mention this in the main text of the modified manuscript (see page 6 of the updated manuscript).

21

In all Figure captions I suggest to add a one sentence description of the main message conveyed at the beginning.

We thank the Referee for this helpful suggestion. In response, we have updated all figure captions to include a brief one-sentence description of the main message.

22

The Appendix may be moved to a supplemental material where more details about the construction of the path integral can be provided, e.g. the action that is employed. Also, some relevant references (even if this means textbooks) would be nice to be provided.

This has been done (see *Supplementary Note 2*). In the "path-integral-like form" for the tunneling amplitude we only divide the time t into M sub-intervals of the length t/M, which is the first step in the usual procedure for rewriting a quantum transition amplitude in path-integral form. Each factor $\langle s_r|e^{-iHt/M}|s_{r-1}\rangle$ is just a transition amplitude for short time t/M, computed using the original Hamiltonian. Therefore, it is not necessary here to take the next step and rewrite the expression using a Lagrangian. To avoid confusion, we have removed reference to a "path integral" and furthermore we now refer to "sequences" of sites visited instead of "paths", again to avoid confusion with classical-like trajectories for which an action would be computed. A textbook citation has been added, and, as the Referee recommended, we took the opportunity to add more detail to the proof.

Referee 2

1

One of the authors conclusions is that the tunneling probability of the bound pair of fermions (doublon) is asymmetric. This implies, in particular, that doublons of cold atoms loaded into the sawtooth optical lattice should exhibit directed current. This would be a wonderful effect but I have never heard of it and I am afraid that it does not exist.

We would like to clarify that we do not claim that the doublon tunnels as a whole. What we actually calculate in our manuscript is the expectation value of $\hat{n}_L = \hat{n}_{L,\uparrow} + \hat{n}_{L,\downarrow}$, which is a one-body operator. However, to account for the mentioned two-body contributions, we have computed the time-averaged $2\langle \hat{n}_{L,\uparrow}\hat{n}_{L,\downarrow}\rangle$ (see Fig. 7). As shown, these expectation values are negligibly small and do not depend on the barrier orientation. This indicates that the tunneling of the doublon as a whole is symmetric.

2

Presented numerical results clearly indicate a possibility for doublon dissociation. Thus, one should distinguish between tunneling and dissociation probabilities.

In accordance with the response to the previous point, the main contribution to $\langle \hat{n}_L \rangle$ comes from the single particle after the doublon dissociation. To prevent any potential misunderstanding, we have explicitly clarified this point in the main text of the manuscript (see page 6 of the modified manuscript).

To support the above statements quantitatively, we have additionally computed the probability of finding a doublon over time by measuring the operator:

$$\hat{n}_{\mathrm{d}} = \sum_{i=1}^{L} \hat{n}_{i,\uparrow}\hat{n}_{i,\downarrow}. \tag{2}$$

Figure 8 shows the evolution of $\langle \hat{n}_{\mathrm{d}} \rangle$ and $\langle \hat{n}_j \rangle$. As seen, at $t \approx 30/J$ the lowest probability of finding a doublon coincides with the highest probability of detecting a particle after the barrier (the very right column of $\langle \hat{n}_j \rangle$). This suggests that doublon dissociation occurs with further tunneling of the single particle. Conversely, the doublon association is observed when the particle tunnels back as indicated by the maximum and minimum values of $\langle \hat{n}_{\mathrm{d}} \rangle$ and $\langle \hat{n}_L \rangle$, respectively.

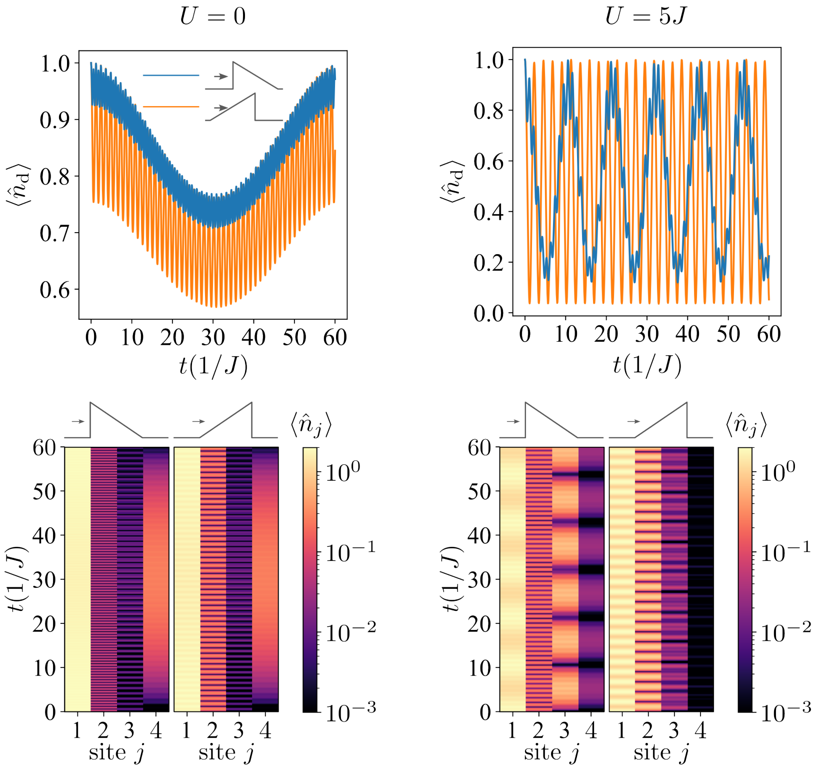

Figure 8: Time-evolution of $\langle \hat{n}_{\mathrm{d}} \rangle$ (top) and $\langle \hat{n}_j \rangle$ (bottom). The system size is $L = 4$ and the height of the barrier is $h = 10J$.

3

Time-averaged quantities may depend on the interval T. There is no comment on this issue in the manuscript.

Fig. 9 illustrates how the time-averaged values of $\langle \hat{n}_{h/2} \rangle$ and $\langle \hat{n}_L \rangle$ depend on the interval T. As one can see, the expectation value of $\langle \hat{n}_{h/2} \rangle$ remains largely unaffected by increasing T. However, $\langle \hat{n}_L \rangle$ exhibits noticeable deviations for small values of U. At this point we would like to emphasize that the primary focus of our study is on the $U \gg J$ regime, exactly where the phenomena of underbarrier resonant trapping and highly-asymmetric resonant tunneling take place. In this regime, where $U \approx 10\ J$, the difference between simulations performed for $T = 100/J$ and $T = 300/J$ is negligibly small.

We have added a comment regarding the robustness of our results with respect to the increase of the interval T in the main text of the manuscript (see page 9 of the modified manuscript).

Referee 3

1

My main criticism of this manuscript lies in Sec. IID, where the singlet and triplet states are introduced. After independently verifying the authors results of Fig. 4, I am confident that their definitions of the triplet and singlet state are reversed. Below I provide the background of understanding why this is the case and then describe the required changes to their manuscript to address this issue.

We sincerely appreciate the Referee's careful verification of our results and their detailed feedback regarding the definitions of the singlet and triplet states in the corresponding section. After reviewing our derivations and comparing them with the Referee's observations, we have carefully corrected the definitions where necessary to ensure consistency throughout the manuscript.

Absolutely, the triplet and singlet states are defined as $\hat{\mathbf{S}}^2|\Psi_t\rangle = \hbar^2 1(1+1)|\Psi_t\rangle$ and $\hat{\mathbf{S}}^2|\Psi_s\rangle = 0\hbar^2|\Psi_s\rangle$, respectively. To define the $\hat{\mathbf{S}}^2$ operator we followed the steps provided by the Referee.

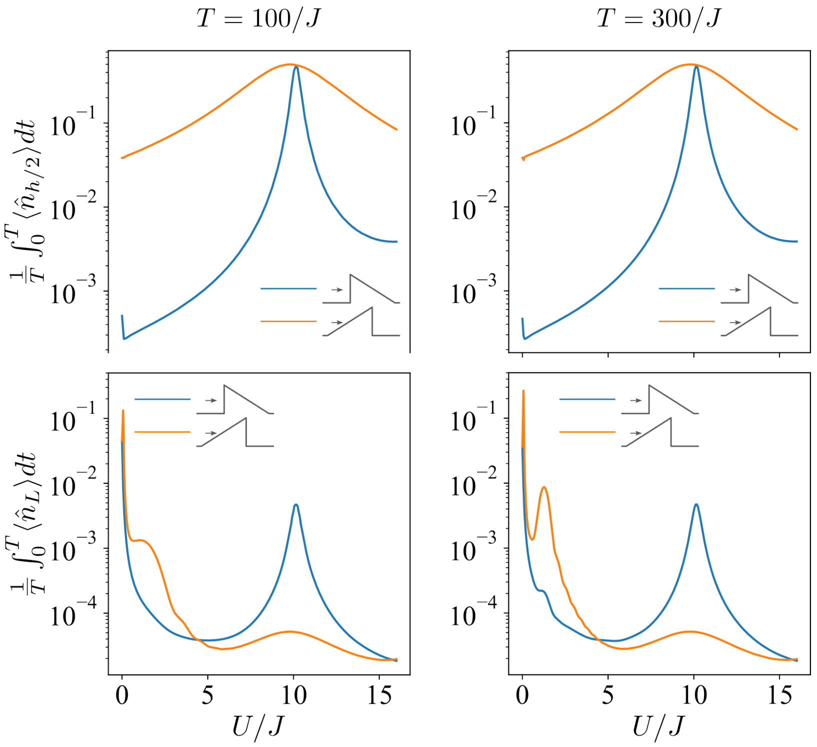

Figure 9: For the $L = 4$ system, the time-averaged expectation value of $\langle \hat{n}_{h/2}\rangle$ (upper row), and $\langle \hat{n}_L\rangle$ (lower row) as a function of the interaction amplitude U (in units of J). The initial state is a doublon positioned at the first site. The barrier height is $h = 20J$. Left: $T = 100/J$; right: $T = 300/J$. For $U = 10J$, the interaction strength coincides with the external potential strength at the site j^*.

To make the above concrete, notice that $\hat{\mathbf{S}}^2$ reads

$$\begin{aligned}\hat{\mathbf{S}}^2 &\equiv \hat{S}_x\hat{S}_x + \hat{S}_y\hat{S}_y + \hat{S}_z\hat{S}_z \\ &= \hat{S}_+\hat{S}_- + \hat{S}_z(\hat{S}_z - 1) \\ &= \hbar^2\left(\hat{N}_\uparrow - \sum_{j,k=1}^{L} \hat{c}^\dagger_{j\uparrow}\hat{c}^\dagger_{k\downarrow}\hat{c}_{k\uparrow}\hat{c}_{j\downarrow}\right) + \hat{S}_z(\hat{S}_z - 1),\end{aligned}$$

with $\hat{N}_\uparrow \equiv \sum_{j=1}^{L}\hat{c}^\dagger_{j\uparrow}\hat{c}_{j\uparrow}$ and the commutation or anticommutation properties of the operators $\hat{c}^{(\dagger)}_{j\uparrow}$ and $\hat{c}^{(\dagger)}_{j\downarrow}$ control whether the system is spinor (single-species spin-1/2 fermions) or pseudo-spinor (two distinguishable fermionic species).

We believe the equation above includes a typo in the second term of the last line. It should read as

$$\hat{\mathbf{S}}^2 = \hbar^2\left(\hat{N}_\uparrow - \sum_{j,k=1}^{L} \hat{c}^\dagger_{j\uparrow}\hat{c}^\dagger_{k\downarrow}\hat{c}_{j\downarrow}\hat{c}_{k\uparrow}\right) + \hat{S}_z(\hat{S}_z - 1), \tag{3}$$

where the annihilation operators $\hat{c}_{j\downarrow}$ and $\hat{c}_{k\uparrow}$ are not yet interchanged, since this would require to employ the commutation relations specified only on the next step.

A nice device for simplifying calculations is to define

$$\hat{c}_{j\uparrow}\hat{c}_{k\downarrow} = \zeta\hat{c}_{k\downarrow}\hat{c}_{j\uparrow}, \quad \hat{c}_{j\uparrow}\hat{c}^{\dagger}_{k\downarrow} = \zeta\hat{c}^{\dagger}_{k\downarrow}\hat{c}_{j\uparrow},$$

with $\zeta = 1$ (commutation) for pseudo-spinors and $\zeta = -1$ (anti-commutation) for spinors, while of course for the same species the standard anticommutation rules hold, i.e. $\{\hat{c}_{j\alpha}, \hat{c}^{\dagger}_{k\alpha}\} = \delta_{j,k}$ and $\{\hat{c}_{j\alpha}, \hat{c}_{k\alpha}\} = 0$ for $\alpha \in \{\downarrow, \uparrow\}$.

We would like to thank the Referee for this insightful suggestion. It has helped us gain a deeper understanding of how the Hamiltonian construction is implemented in the QuSpin package.

Taking into account the form of $\hat{\mathbf{S}}^2$ operator as in Eq. (3), we verified that the doublon state $|\Psi_{k,\mathrm{d}}\rangle = \hat{c}^{\dagger}_{k\uparrow}\hat{c}^{\dagger}_{k\downarrow}|0\rangle$ is an eigenstate of $\hat{\mathbf{S}}^2$ with $\hat{S}^2|\Psi_{\mathrm{d}}\rangle = 0$.

To understand where these discrepancies originate, consider the states defined in the main text:

$$|\Psi_t\rangle = \frac{1}{\sqrt{2}}\left[\hat{c}^{\dagger}_{1\uparrow}\hat{c}^{\dagger}_{2\downarrow} + \hat{c}^{\dagger}_{1\downarrow}\hat{c}^{\dagger}_{2\uparrow}\right]|0\rangle, \tag{4}$$

$$|\Psi_s\rangle = \frac{1}{\sqrt{2}}\left[\hat{c}^{\dagger}_{1\uparrow}\hat{c}^{\dagger}_{2\downarrow} - \hat{c}^{\dagger}_{1\downarrow}\hat{c}^{\dagger}_{2\uparrow}\right]|0\rangle. \tag{5}$$

It can be easily verified that $\hat{S}^2|\Psi_t\rangle = \hbar^2(1-\zeta)|\Psi_t\rangle$ and $\hat{S}^2|\Psi_s\rangle = \hbar^2(1+\zeta)|\Psi_s\rangle$, and therefore in the pseudo-spinor case $\zeta = 1$ the singlet and triplet character of the states is reversed.

Indeed, we verified that the action of $\hat{\mathbf{S}}^2$ on the states in Eqs. (4), (5) matches the expressions provided by the Referee. We appreciate this valuable discussion and agree that in the pseudo-spinor case, the singlet and triplet character of the states was reversed.

By examining the matrix form of the Hamiltonian generated by the QuSpin package, we see that the off-diagonal matrix elements do not consider a factor $(-1)^{\sum_{l=1}^{m-1} n_l}$. This indicates that QuSpin operates in terms of pseudo-spinors. To further validate this, we ran additional simulations using our alternative code, which explicitly accounts for the fermionic sign. These independent checks confirm that in the spinor case the triplet state reveals symmetric tunneling, whereas the initial singlet state results in the asymmetric tunneling.

Corrections After the authors verify the above they need to amend the discussion on the paper, since now the spin-triplet states preserve the tunneling symmetry and the spin-singlets show strong asymmetry which is the other way around from what they write in their abstract and main text. In addition, it might be useful to include a brief discussion about the $\hat{\mathbf{S}}^2$ symmetry. On a related note, it would also be nice that the authors give a brief motivation on why they use a six site system in Sec. IID, which is of course in order to define triplet states, which are impossible for the four site system.

After verifying the spin character of the states, we acknowledge that the spin-triplet states preserve tunneling symmetry, while the spin-singlet states exhibit asymmetry, which is the opposite of our original claim. Despite the fact that QuSpin operates in terms of pseudo-spinors, we believe that working with spinors is more conventional for readers. Therefore, we have decided to maintain the definitions of singlet and triplet states as presented in the main manuscript. Nevertheless, we have revised the text to clearly state that the singlet state leads to asymmetric tunneling, while the triplet state preserves the symmetry (see section *Results* subsection *Symmetry considerations* on page 8).

Additionally, we have included a brief discussion on the $\hat{\mathbf{S}}^2$ symmetry, emphasizing that the external potential, hopping, and interaction terms are spin-independent, ensuring that $\hat{\mathbf{S}}^2$ remains invariant under SU(2) spin rotations (see section *Results* subsection *Model* on page 5).

Furthermore, following the Referee's suggestion, we have explicitly clarified (see section *Results* subsection *Symmetry considerations* on page 8) the motivation behind using a six-site system.

2

The second point I would like to see improved is the introduction of the manuscript. Currently I think that it does appeal to people interested in ultracold atoms, however, I think that it can be framed in a a sense that is more appealing to a wider audience. In particular, while the tunneling is indeed important in a large variety of physical systems and the authors results can be measured in ultracold experiments, I think it should be more stressed that controlling tunneling is important for applications such as the ones mentioned in the last paragraph of the introduction and also that controlling tunneling dynamics by spin is a very attractive feature, as it provides an interface towards proposed quantum devices that employ (pseudo-)spin manipulation.

We appreciate the Referee's feedback and have taken steps to make the introduction more appealing to a broader audience. In the revised version (see page 4 of the modified manuscript), we emphasize not only the relevance of tunneling phenomena in ultracold atom systems but also highlight its importance for a wide range of technological applications.

3

I think that section IIC does not really add much to the discussion since it is focussed on the case of extremely small mobility for one of the spin-states. The authors can verify that in this case the total spin of Eq. (3) is not a symmetry of the Hamiltonian and thus triplet and singlet states are admixed which cannot happen in any other scenario the authors have considered. For this reason I would propose that this section is transferred to a separate publication that maybe examines how the triplet and singlet states couple when the $\hat{S}^2$ symmetry of the system is lifted, if the authors wish to examine this case.

We agree that this section focuses on the case of extremely small mobility for one of the spin states. In light of this, we have decided to move it to the supplementary materials (see *Supplementary Note 1*). We hope that this adjustment improves the focus of the main manuscript and appreciate the Referee's suggestion.

4

In Sec. IIA the Landau theorem on the symmetry of tunneling is mentioned and then IIB it is resolved that this does not apply to the author's system. I think that the authors should point out the limitations of this theorem already at Sec. IIA and then mention that they prove its extension for their system in IIB and Appendix A. On a related note in Sec. IIB it might be helpful to describe in a couple of words the main idea behind the derivation of each theorem.

We thank the Referee for their suggestion. To clarify the limitations of Landau's theorem, we have revised the corresponding section to explicitly state that the theorem applies to continuous systems in infinite space and requires significant modifications for discrete finite systems (see page 4 of the revised manuscript).

In section *Results* (subsection *Noninteracting fermions*, see page 6) we have also included a brief explanation of the main idea behind our derivation.

5

In Sec. IIB before the simplified statement of the theorem there is a typo "the two curves agree overlap perfectly", I would remove "agree" here.

We thank the Referee for pointing out this typo. We have removed the word "agree" from the sentence in the corresponding section.

TITLE: Few-fermion resonant tunneling and underbarrier trapping in asymmetric potentials
AUTHORS: Elvira Bilokon, Valeriia Bilokon, Dusty R. Lindberg, Andrii Sotnikov, Lev Kaplan, and Denys I. Bondar

We thank the Referees for their thoughtful comments and valuable suggestions, which have helped us improve the manuscript. Our detailed responses to their points are provided below. Modified (or new) parts of the text have been marked in green in the PDF file.

Summary of main modifications:

1. **Introduction:**
   - We have expanded the reference list. (R2.2; R3.2)
2. **Results:**
   - We have clarified how many-body resonant tunneling differs from conventional single-particle resonant tunneling. (R1.1)
   - We have replaced the right panel in Fig. 5 of the manuscript with the visualization for $L = 20$ sites. (R2.1)
3. **Supplementary Material:**
   - We have added *Supplementary Note 3* to show how time to reach site j^* scales with system size. (R1.4)

Responses to the Referee comments

Referee 1

1

Explicitly mention why and how the single-particle tunneling processes, such as the many-body resonant tunneling, discussed in the main text are different from the previously known single-particle resonant tunneling. I think this distinction should be made clear in order to ease the understanding of the reader, especially the non-expert one.

In response to this recommendation, we have added the clarification in the modified manuscript (see page 12). Namely, we say:

In conclusion, we reiterate that the phenomenon of many-body quantum tunneling has no single-particle analogue. In the single-particle case, the resonant tunneling cannot occur for a single barrier, requiring two or more barriers. In contrast, our many-body resonant tunneling occurs in a single barrier and arises from the interplay between inter-particle interactions and the external potential.

2

A natural question that emerges is whether any of the underbarrier resonant trapping or many-body resonant tunneling have classical analogues. Can the authors comment on this issue?

We appreciate the Referee's insightful question about possible classical analogues to the underbarrier resonant trapping and many-body resonant tunneling phenomena described in our work. To the best of our knowledge, effects presented in our work are purely quantum in nature and do not have direct classical analogues.

3

Mention the degree of convergence of the time-averaged probabilities (number operator) with respect to the considered evolution time. Currently, the statement that the relevant observables are largely unaffected by increasing the final time T is a bit general.

To demonstrate the degree of convergence, we have calculated the relative difference between time-averaged expectation values for different evolution times ($T = 100/J$ and $T = 300/J$). Specifically, we define

$$\Delta^j_{\rm rel} = \frac{|\langle \hat{n}_j \rangle^{T=300}_{\rm avg} - \langle \hat{n}_j \rangle^{T=100}_{\rm avg}|}{\langle \hat{n}_j \rangle^{T=300}_{\rm avg}}. \quad (1)$$

The dependence $\Delta^j_{\rm rel}(U)$ for $j = \{h/2, L\}$ is shown in Fig. 1. For the underbarrier resonant trapping

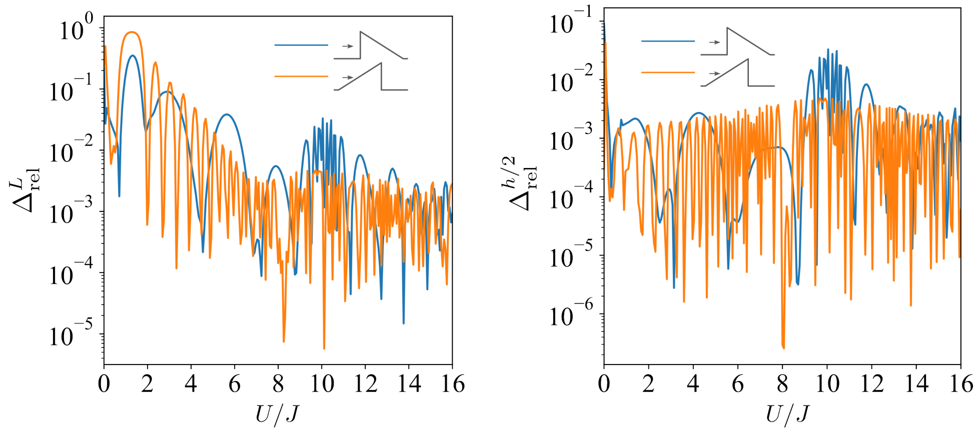

Figure 1: Relative difference $\Delta^j_{\rm rel}$ of time-averaged $\langle \hat{n}_L \rangle$ (left) and $\langle \hat{n}_{h/2} \rangle$ (right) as a function of the interaction strength U. The height of the barrier $h = 20J$.

regime ($U \approx 10J$), which is our primary focus, we find that both $\Delta_{\rm rel}^{L}$ and $\Delta_{\rm rel}^{h/2}$ are less than 3.5%.

4

It would be nice to see a scaling of the time required to reach site j* with respect to the system size for the underbarrier resonant trapping. This will assist the experimental realization of this phenomenon.

We thank the Referee for raising this question. We have added a *Supplementary Note 3* to demonstrate how the time required to reach site j^* scales with the system size.

5

Please check carefully the entire manuscript for typographical errors.

The revised manuscript has been carefully checked by all co-authors to minimize the possibility of any remaining typographical errors.

Referee 2

1

To bring this toy model closer to laboratory experiments with cold atoms (to which the authors constantly refer to) one should consider larger lattices, see remark No.18 of the first Reviewer. In this respect I would recommend substituting the right panel in Fig.5 in the manuscript for the right panel in Fig.5 in the response letter, where one can see how the dublon hits the barrier.

We thank the Referee for this constructive suggestion. Following their recommendation, we have replaced the right panel in Fig. 5 of the manuscript with the visualization for $L = 20$ sites that was previously shown in Fig. 5 of our response letter.

2

And, of course, the work by Kolovsky, et al. of 2012 has to be cited because it discusses the same phenomenon of the resonant trapping but for the pair of interacting bosons. (Please, also check the recent paper "Two-particle tunneling and the impact of interaction" by Brugger, Dittel, and Buchleitner published in Physical Review A.)

We apologize for simply overlooking the work by Kolovsky et al. (2012) which the Referee kindly pointed out in their previous report. We have now added this reference, along with the recent paper by Brugger, Dittel, and Buchleitner, to our manuscript.

Referee 3

1

Referee question 2.3 raised a valid point regarding the convergence of the time average of the observables the authors present in their manuscript. The authors reply this question with figure 9 of the referee report. Since the authors have access to the full eigenspectrum, this referee can propose an alternative method for evaluating the time-averaged value of observables, that the authors might consider useful.

We thank the Referee for providing us with the alternative method to evaluate time-averaged observables, which would eliminate concerns about convergence. We appreciate this valuable suggestion and will consider it in our later studies.

2

The authors might find that the follow up work of New J. Phys. **22**, 063058 (2020), Phys. Rev. Research **5**, 043039 (2023) also interesting for them.

We thank the Referee for these additional references. The first paper (New J. Phys. 22, 063058 (2020)) was already cited in our manuscript. We have now added the more recent work (Phys. Rev. Research 5, 043039 (2023)) to our reference list.

Referee Report on: COMMSPHYS-25-0167-T

In the manuscript titled "Few-fermion resonant tunneling and underbarrier trapping in asymmetric potentials" the authors investigate small fermionic two-component mixtures in few-site asymmetric potentials in both the interacting an non-interacting case. The first central finding of the authors is that despite the potential breaks the reflection symmetry of the system, whether the exhibited tunneling dynamics of the system is symmetric or not, instead depends on the spin state of the system. The second key finding is that bound states within the potential can be populated during the dynamics of a singlet state, which can be interrogated by another particle to generate resonant tunneling. I find that the author's results bring to the attention of the ultracold atom community important features of tunneling dynamics which are overlooked and maybe the ideas provided here can be useful for applications even to other fields. The manuscript is well-written and I would support its publication in Communications Physics after a substantial revision.

In the following part, I elaborate on the main points that should be addressed in the revised manuscript in order of severity and provide some additional comments for possible improvements.

Main points

A. My main criticism of this manuscript lies in Sec. IID, where the singlet and triplet states are introduced. After independently verifying the authors results of Fig. 4, I am confident that their definitions of the triplet and singlet state are reversed. Below I provide the background of understanding why this is the case and then describe the required changes to their manuscript to address this issue.

Impact of total spin: First notice that the singlet and triplet states should be defined such as $\hat{S}^2|\Psi_t\rangle = \hbar^2 1(1+1)|\Psi_t\rangle$ for the triplet state and $\hat{S}^2|\Psi_s\rangle = 0\hbar^2|\Psi_t\rangle$ for the singlet state. However, the main issue is how do we define $\hat{S}^2$? The authors can straightforwardly verify that the intuitive definitions

$$\hat{S}_\mu \equiv \frac{\hbar}{2}\sum_{j=1}^{L}\sum_{\alpha,\beta\in\{\uparrow,\downarrow\}} \hat{c}^\dagger_{j\alpha}\sigma^\mu_{\alpha\beta}\hat{c}_{j\beta} \tag{1}$$

with $\hat{c}^{(\dagger)}_{j\alpha}$ the annihillation (creation) operator destroying (creating) a fermion with spin $\alpha \in \{\uparrow,\downarrow\}$ in the site $j = 1, 2, \ldots, L$ and $\sigma^\mu_{\alpha\beta}$ the matrix elements of the $\mu \in \{x, y, z\}$ Pauli matrix, when supplemented by

$$\begin{aligned}\hat{S}_+ &\equiv \hat{S}_x + i\hat{S}_y = \hbar\sum_{j=1}^{L}\hat{c}^\dagger_{j\uparrow}\hat{c}_{j\downarrow},\\ \hat{S}_- &\equiv \hat{S}_x - i\hat{S}_y = \hbar\sum_{j=1}^{L}\hat{c}^\dagger_{j\downarrow}\hat{c}_{j\uparrow},\end{aligned} \tag{2}$$

lead to the well-behaving definitions of $\hat{\mathbf{S}}^2$ (i.e. satisfying the appropriate commutation properties of the spin and its projections), irrespectively on whether the operators corresponding to different spin commute or anticommute. To make the above concrete, notice that $\hat{\mathbf{S}}^2$ reads

$$\begin{aligned}\hat{S}^2 &\equiv \hat{S}_x\hat{S}_x + \hat{S}_y\hat{S}_y + \hat{S}_z\hat{S}_z\\ &= \hat{S}_+\hat{S}_- + \hat{S}_z(\hat{S}_z - 1)\\ &= \hbar^2\left(\hat{N}_\uparrow - \sum_{j,k=1}^{L}\hat{c}^\dagger_{j\uparrow}\hat{c}^\dagger_{k\downarrow}\hat{c}_{k\uparrow}\hat{c}_{j\downarrow}\right) + \hat{S}_z(\hat{S}_z - 1),\end{aligned} \tag{3}$$

with $\hat{N}_\uparrow \equiv \sum_{j=1}^{L}\hat{c}^\dagger_{j\uparrow}\hat{c}_{j\uparrow}$ and the commutation or anticommutation properties of the operators $\hat{c}^{(\dagger)}_{j\uparrow}$ and $\hat{c}^{(\dagger)}_{j\downarrow}$ control whether the system is spinor (single-species spin-1/2 fermions) or pseudo-spinor (two distinguishable fermionic species). A nice device for simplifying calculations is to define

$$\hat{c}_{j\uparrow}\hat{c}_{k\downarrow} = \zeta\hat{c}_{k\downarrow}\hat{c}_{j\uparrow}, \qquad \hat{c}_{j\uparrow}\hat{c}^\dagger_{k\downarrow} = \zeta\hat{c}^\dagger_{k\downarrow}\hat{c}_{j\uparrow}, \tag{4}$$

with $\zeta = 1$ (commutation) for pseudo-spinors and $\zeta = -1$ (anti-commutation) for spinors, while of course for the same species the standard anticommutation rules hold, i.e. $\{\hat{c}_{j\alpha}, \hat{c}^\dagger_{k\alpha}\} = \delta_{j,k}$ and $\{\hat{c}_{j\alpha}, \hat{c}_{k\alpha}\} = 0$ for $\alpha \in \{\downarrow, \uparrow\}$. Finally, it is also straightforward to show that $\hat{S}^2$ as defined above commutes with the Hamiltonian of Eq. (1) in the main-text and thus is a symmetry of the system.

By using the last form of Eq. (3) the action of $\hat{S}^2$ of any many-body state with given particle number in the spin-$\uparrow$ and spin-$\downarrow$ can be easily calculated as the only non-trivial operator essentially exchanges the position of each pair of spin-$\uparrow$ and spin-$\downarrow$ atoms. With these in mind it is very easy to show that the doublon state $|\Psi_{k,\mathrm{d}}\rangle = \hat{c}^\dagger_{k\downarrow}\hat{c}^\dagger_{k\uparrow}|0\rangle$ is an eigenstate of $\hat{S}^2$ with $\hat{S}^2|\Psi_d\rangle = 0$. Notice that since $[\hat{\mathcal{H}}, \hat{S}^2] = 0$ then this state can only couple to singlet state during the dynamics since the total spin should be conserved. However, the authors can verify that what they claim as the triplet state during its time-evolution has finite overlap to the doublon states, which is also noted in the Eq. (6) of the main text. In addition, as obvious from Fig. 6, the tunneling dynamics of an initial doublon state is modified when the doublon is initialized on a different side of the barrier contradicting the authors claim that singlet states lead to symmetric tunneling. To understand where these discrepancies originate, consider the states defined in the main-text

$$\begin{aligned} |\Psi_t\rangle &= \frac{1}{\sqrt{2}}\left[\hat{c}^\dagger_{1\uparrow}\hat{c}^\dagger_{2\downarrow} + \hat{c}^\dagger_{1\downarrow}\hat{c}^\dagger_{2\uparrow}\right]|0\rangle, \\ |\Psi_s\rangle &= \frac{1}{\sqrt{2}}\left[\hat{c}^\dagger_{1\uparrow}\hat{c}^\dagger_{2\downarrow} - \hat{c}^\dagger_{1\downarrow}\hat{c}^\dagger_{2\uparrow}\right]|0\rangle. \end{aligned} \tag{5}$$

It can be easily verified that $\hat{S}^2|\Psi_t\rangle = \hbar^2(1-\zeta)|\Psi_t\rangle$ and $\hat{S}^2|\Psi_s\rangle = \hbar^2(1+\zeta)|\Psi_t\rangle$ and therefore in the pseudo-spinor case $\zeta = 1$ the singlet and triplet character of the states is reversed.

The final issue that we need to address is how to understand from a given computer program whether it considers the spinor or the pseudo-spinor case? The answer lies in examining how a creation and annihilation operator acts on the many-body number state that many-body codes typically use. Recall, that the creation or annihillation of a fermionic particle at a state m-th single particle state of the number state invokes a factor $(-1)^{\sum_{l=1}^{m-1} n_l}$. As a consequence, the off-diagonal matrix elements of the Hamiltonian e.g. stemming from the term $\propto J$ are affected by these factors. The authors need to verify whether the corresponding sum in these factors includes (spinor) or excludes (pseudo-spinor) states with the opposite spin than the operator applied.

Corrections After the authors verify the above they need to amend the discussion on the paper, since now the spin-triplet states preserve the tunneling symmetry and the spin-singlets show strong asymmetry which is the other way around from what they write in their abstract and main text. In addition, it might be useful to include a brief discussion about the $\hat{S}^2$ symmetry. On a related note, it would also be nice that the authors give a brief motivation on why they use a six site system in Sec. IID, which is of course in order to define triplet states, which are impossible for the four site system.

B. The second point I would like to see improved is the introduction of the manuscript. Currently I think that it does appeal to people interested in ultracold atoms, however, I think that it can be framed in a a sense that is more appealing to a wider audience. In particular, while the tunneling is indeed important in a large variety of physical systems and the authors results can be measured in ultracold experiments, I think it should be more stressed that controlling tunneling is important for applications such as the ones mentioned in the last paragraph of the introduction and also that controlling tunneling dynamics by spin is a very attractive feature, as it provides an interface towards proposed quantum devices that employ (pseudo-)spin manipulation.

C. I think that section IIC does not really add much to the discussion since it is focussed on the case of extremely small mobility for one of the spin-states. The authors can verify that in this case the total spin of Eq. (3) is not a symmetry of the Hamiltonian and thus triplet and singlet states are admixed which cannot happen in any other scenario the authors have considered. For this reason I would propose that this section is transferred to a separate publication that

maybe examines how the triplet and singlet states couple when the $\hat{S}^2$ symmetry of the system is lifted, if the authors wish to examine this case.

With these corrections in place I think a revised manuscript would be a valuable contribution to Communications Physics. Below I provide a few additional minor comments that could possibly help the authors to polish their manuscript further.

Minor comments

1. In Sec. IIA the Landau theorem on the symmetry of tunneling is mentioned and then IIB it is resolved that this does not apply to the author's system. I think that the authors should point out the limitations of this theorem already at Sec. IIA and then mention that they prove its extension for their system in IIB and Appendix A. On a related note in Sec. IIB it might be helpful to describe in a couple of words the main idea behind the derivation of each theorem.

2. In Sec. IIB before the simplified statement of the theorem there is a typo "the two curves agree overlap perfectly", I would remove "agree" here.

Second Referee Report on: COMMSPHYS-25-0167-T

The referees have addressed my comments in a satisfactory manner in their revised manuscript and I would support its publication in Communications Physics. Below there are some minor comments that the authors might find useful for a final polish of their manuscript before publication.

Minor comments

A. Referee question 2.3 raised a valid point regarding the convergence of the time average of the observables the authors present in their manuscript. The authors reply this question with figure 9 of the referee report. Since the authors have access to the full eigenspectrum, this referee can propose an alternative method for evaluating the time-averaged value of observables, that the authors might consider useful. Starting from the time evolution of the total wavefunction we have

$$|\Psi(t)\rangle = \sum_n \langle \Phi_n|\Psi(0)\rangle e^{-iE_n t/\hbar}|\Phi_n\rangle, \tag{1}$$

where $|\Phi_n\rangle$ and E_n are the n-th eigenstate and eigenenergy of $\hat{\mathcal{H}}$ of Eq. (1) respectively. With this expression it can be proved easily that for every real symmetric operator $\hat{O}$

$$\begin{aligned}\langle \Psi(t)|\hat{O}|\Psi(t)\rangle = &\underbrace{\sum_n |\langle \Phi_n|\Psi(0)\rangle|^2 \langle \Phi_n|\hat{O}|\Phi_n\rangle + \sum_{\{n<m|E_n=E_m\}} A_{n,m}\langle \Phi_n|\hat{O}|\Phi_m\rangle \cos\phi_{n,m}}_{\equiv\langle \hat{O}\rangle_{\text{static}}} \\ &+ \underbrace{\sum_{\{n<m|E_n\neq E_m\}} A_{n,m}\langle \Phi_n|\hat{O}|\Phi_m\rangle \cos\left(\frac{E_n-E_m}{\hbar}t+\phi_{n,m}\right)}_{\equiv\langle \hat{O}(t)\rangle_{\text{fluc}}},\end{aligned} \tag{2}$$

where I have defined

$$\begin{aligned}A_{n,m} &= 2|\langle \Phi_n|\Psi(0)\rangle^* \langle \Phi_m|\Psi(0)\rangle|, \\ \phi_{n,m} &= \arg\left(\langle \Phi_n|\Psi(0)\rangle^* \langle \Phi_m|\Psi(0)\rangle\right).\end{aligned} \tag{3}$$

Based on this expansion it can be proven that the time-averaging integral of $\langle \hat{O}(t)\rangle_{\text{fluc}}$ decays $\frac{1}{T}\int_0^T \mathrm{d}t\ \langle \hat{O}(t)\rangle_{\text{fluc}} \propto T^{-1}$, where T is the total integration time. Thus the time-averaged expectation value of the operator $\hat{O}$ for $T \to \infty$ is equal to $\langle \hat{O}\rangle_{\text{static}}$.

B. The authors might find that the follow up work of New J. Phys. **22**, 063058 (2020), Phys. Rev. Research **5**, 043039 (2023) also interesting for them.
