# Supplementary material for: Few-fermion resonant tunneling and underbarrier trapping in asymmetric potentials
Source: Commun Phys. 2025 Jun 20;8(1):259. doi: 10.1038/s42005-025-02189-9 (PMC12181076; doi:10.1038/s42005-025-02189-9)
Supplement: Supplementary file 2 — Transparent Peer Review file [file 42005_2025_2189_MOESM2_ESM.pdf]

Supplementary Information for "Few-fermion resonant tunneling and underbarrier trapping in asymmetric potentials"

Elvira Bilokon,[1,2,*] Valeriia Bilokon,[1,2] Dusty R. Lindberg,[1]
Lev Kaplan,[1] Andrii Sotnikov,[2,3] and Denys I. Bondar [1,†]

[1]*Department of Physics and Engineering Physics, Tulane University, New Orleans, Louisiana 70118, United States*
[2]*Akhiezer Institute for Theoretical Physics, NSC KIPT, Akademichna 1, 61108 Kharkiv, Ukraine*
[3]*Karazin Kharkiv National University, Svobody Square 4, 61022 Kharkiv, Ukraine*

Supplementary Note 1: INTERACTING FERMIONS: THE FALICOV–KIMBALL LIMIT

As found, noninteracting particles undergo symmetric tunneling regardless of the barrier orientation. However, the system evolution changes significantly as soon as the interaction U between fermions is included. To illustrate this, we consider the case of an immobile particle, which corresponds to the Falicov-Kimball limit. The Falicov-Kimball model [1, 2] arises in condensed matter physics to describe correlated electron systems, particularly metal-insulator transitions in compounds with both itinerant and localized electrons. Similar to our model, the itinerant particles do not interact directly with each other but experience an interaction with the localized particles that are bound to specific locations in the lattice. This interaction can effectively act as a site-dependent potential barrier that influences the movement of itinerant electrons. With only one spin component allowed to propagate, the Falicov-Kimball model becomes simpler than the Hubbard model and is exactly solvable in the limit of infinite spatial dimensions [3, 4].

The Fermi–Hubbard Hamiltonian that describes our model reads

$$\hat{\mathcal{H}} = -J \sum_{j=1,\sigma=\uparrow,\downarrow}^{L-1} \left(\hat{c}^{\dagger}_{j,\sigma}\hat{c}_{j+1,\sigma} + \hat{c}^{\dagger}_{j+1,\sigma}\hat{c}_{j,\sigma} \right) + \sum_{j=1}^{L} (U\hat{n}_{j,\uparrow}\hat{n}_{j,\downarrow} + V_j^{\text{ex}}\hat{n}_j) \tag{1}$$

with site-dependent asymmetric external potential V_j^{ex} defined as

$$V_j^{\text{ex (a)}} = \begin{cases} h, & \text{if } j = L/2; \\ h/2, & \text{if } j = L/2+1; \\ 0, & \text{otherwise;} \end{cases}$$

$$\text{or} \tag{2}$$

$$V_j^{\text{ex (b)}} = \begin{cases} h/2, & \text{if } j = L/2; \\ h, & \text{if } j = L/2+1; \\ 0, & \text{otherwise.} \end{cases}$$

In our case, a fermion with $\sigma =\downarrow$ acts as a localized particle. Since it remains confined to the first site, we only need to track the position of the itinerant fermion with $\sigma =\uparrow$. As a result, the Hamiltonian from Supplementary Figure (1) becomes

$$\hat{\mathcal{H}} = -J \sum_{j=1}^{L-1} \left(\hat{c}^{\dagger}_{j,\uparrow}\hat{c}_{j+1,\uparrow} + \hat{c}^{\dagger}_{j+1,\uparrow}\hat{c}_{j,\uparrow} \right) + \sum_{j=1}^{L} (U\hat{n}_{j,\uparrow}\delta_{j,1} + V_j^{\text{ex}}\hat{n}_{j,\uparrow}) \,. \tag{3}$$

Explicitly, for the case when the particles face the vertical side of the barrier [see Supplementary Figure 1(a)], $V_{L/2}^{\text{ex}} = V_2^{\text{ex}} = h$ and $V_{L/2+1}^{\text{ex}} = V_3^{\text{ex}} = h/2$. This leads to the following Hamiltonian

$$\hat{\mathcal{H}}_a = -J \sum_{j=1}^{L-1} \left(\hat{c}^{\dagger}_{j,\uparrow}\hat{c}_{j+1,\uparrow} + \hat{c}^{\dagger}_{j+1,\uparrow}\hat{c}_{j,\uparrow} \right) + U\hat{n}_{1,\uparrow} + h\hat{n}_{2,\uparrow} + \frac{h}{2}\hat{n}_{3,\uparrow} \,. \tag{4}$$

In the second case [see Supplementary Figure 1(b)], when particles are positioned before the angled side of the barrier, $V_{L/2}^{\text{ex}} = V_2^{\text{ex}} = h/2$ and $V_{L/2+1}^{\text{ex}} = V_3^{\text{ex}} = h$. Then Supplementary Equation (3) transforms into

$$\hat{\mathcal{H}}_b = -J \sum_{j=1}^{L-1} \left(\hat{c}^{\dagger}_{j,\uparrow}\hat{c}_{j+1,\uparrow} + \hat{c}^{\dagger}_{j+1,\uparrow}\hat{c}_{j,\uparrow} \right) + U\hat{n}_{1,\uparrow} + \frac{h}{2}\hat{n}_{2,\uparrow} + h\hat{n}_{3,\uparrow} \,. \tag{5}$$

It is clear from Supplementary Equations (4) and (5) that the inter-particle interaction U effectively changes the external potential by altering the local energy of the site when a spin-up particle is present. As one can see from Supplementary Figure 1, the modified effective barriers, depicted by dashed lines, are not mirror images of each other. Thus, the asymmetry in $\langle \hat{n}_L \rangle$ is caused by penetration of the spin-↑ particle through two different barriers.

Supplementary Note 2: PROOF OF THE SYMMETRY OF TUNNELING PROBABILITY IN THE ABSENCE OF INTERACTION

* ebilokon@tulane.edu
† dbondar@tulane.edu

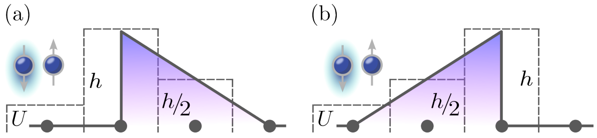

Supplementary Figure 1. **Initial configurations in the Falicov-Kimball limit.** Within Falicov-Kimball limit, spin-↓ and spin-↑ fermions represent the localized and itinerant particles, respectively. The interaction U induces an effective modification of the external potential V_j^{ex}. The modified effective barrier is depicted by dashed lines.

Theorem 1. *Let us assume*

1. *The Fermi-Hubbard Hamiltonian $\hat{\mathcal{H}}$ from Supplementary Equation (1) with no interaction $U = 0$ with the external potential V_j^{ex} schematically depicted in Supplementary Figure 2; $V_j^{\mathrm{ex}} = 0$ for j in regions A and C, whereas V_j^{ex} is arbitrary in region B.*

2. *The probability of a particle tunneling into region C is*

$$\langle \hat{n}_C(t) \rangle = \langle \Psi(t) | \, \hat{n}_C \, | \Psi(t) \rangle \, , \qquad \hat{n}_C = \sum_{c=L_A+L_B+1}^{L} \hat{n}_c, \tag{6}$$

where the wavefunction $|\Psi(t)\rangle = e^{-it\hat{\mathcal{H}}} |\Psi(0)\rangle$ describes evolution of the initial state $|\Psi(0)\rangle$ of a single particle positioned in region A, i.e.,

$$|\Psi(0)\rangle = \sum_{a=1}^{L_A} \kappa_a \hat{c}_a^\dagger |0\rangle, \qquad \sum_{a=1}^{L_A} |\kappa_a|^2 = 1. \tag{7}$$

3. *The probability of a particle tunneling into region A is*

$$\langle \hat{n}_A(t) \rangle = \left\langle \widetilde{\Psi}(t) \middle| \, \hat{n}_A \, \middle| \widetilde{\Psi}(t) \right\rangle , \qquad \hat{n}_A = \sum_{a=1}^{L_A} \hat{n}_a, \tag{8}$$

where the wavefunction $\left| \widetilde{\Psi}(t) \right\rangle = e^{-it\hat{\mathcal{H}}} \left| \widetilde{\Psi}(0) \right\rangle$ describes evolution of the initial state $\left| \widetilde{\Psi}(0) \right\rangle$ which is the mirror reflection of $|\Psi(0)\rangle$, i.e.,

$$\left| \widetilde{\Psi}(0) \right\rangle = \sum_{a=1}^{L_A} \kappa_a \hat{c}_{L-a+1}^\dagger |0\rangle. \tag{9}$$

While $|\Psi(0)\rangle$ is localized in region A, the initial state $\left| \widetilde{\Psi}(0) \right\rangle$ is localized in region C.

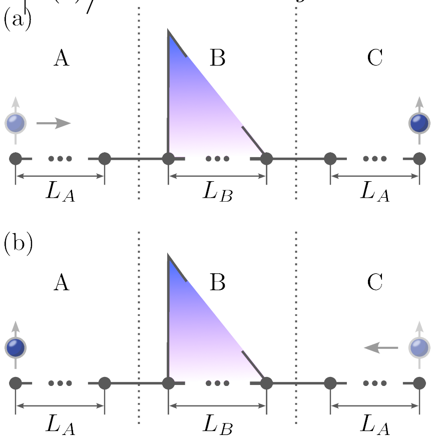

Supplementary Figure 2. **System partition and the initial position of the particle.** For an arbitrary system size, the chain can be divided into three parts: A – the region before the barrier, B – the region with an asymmetric barrier, and C – the region after the barrier. The number of sites before and after the barrier is L_A, whereas L_B is the number of sites under the action of the external potential. The particle is initially positioned (a) from the vertical side, and (b) from the angled side of the barrier. The gray arrow indicates the tunneling direction.

Under these assumptions,

$$\langle \hat{n}_C(t) \rangle = \langle \hat{n}_A(t) \rangle \text{ for all times } t. \tag{10}$$

In other words, the probability of tunneling from left to right equals the probability of tunneling from right to left for a single noninteracting particle initially localized before an arbitrary (including asymmetric) barrier.

Proof. For a single particle, the Hubbard Hamiltonian $\hat{\mathcal{H}}$ acts simply as an $L \times L$ matrix H, with

$$H_{i,j} = V_i^{\mathrm{ex}} \delta_{i,j} - J\delta_{i,j+1} - J\delta_{i,j-1} \, , \tag{11}$$

and as indicated above V_i^{ex} vanishes in regions A and C. For the following argument, the essential properties of H are that (i) H is real, (ii) the restriction of H to region C is identical, after reflection, to the restriction of H to region A, and (iii) only nearest-neighbor hopping is present, so that there is a single coupling linking regions A and B and a single (identical) coupling linking regions C and B.

We start with an amplitude $\langle c|e^{-iHt}|a\rangle$, which we want to relate to $\langle L+1-c|e^{-iHt}|L+1-a\rangle$, where $|a\rangle$ and $|c\rangle$ are in region A and C, respectively (and $|L+1-a\rangle$ and $|L+1-c\rangle$ are in region C and A, respectively). We first split up the total time interval of duration t into M time steps of size t/M and insert a complete set of states after each step [5]:

$$\langle c|e^{-iHt}|a\rangle = \sum_{s_1=1}^{L}\cdots\sum_{s_{M-1}=1}^{L}\langle c|e^{-iHt/M}|s_{M-1}\rangle\langle s_{M-1}|e^{-iHt/M}|s_{M-2}\rangle\cdots\langle s_2|e^{-iHt/M}|s_1\rangle\langle s_1|e^{-iHt/M}|a\rangle$$
$$= \sum_{s_1=1}^{L}\cdots\sum_{s_{M-1}=1}^{L}\prod_{r=1}^{M}\langle s_r|e^{-iHt/M}|s_{r-1}\rangle\,, \tag{12}$$

where $|s_0\rangle = |a\rangle$ and $|s_M\rangle = |c\rangle$. We may rewrite this as a sum over sequences $(a, s_1, s_2, \cdots s_{M-1}, c)$:

$$\langle c|e^{-iHt}|a\rangle = \sum_{(s_0=a,s_1,s_2,\cdots s_{M-1},s_M=c):\ 1\le s_j\le L}\ \prod_{r=1}^{M}\langle s_r|e^{-iHt/M}|s_{r-1}\rangle\,. \tag{13}$$

Each sequence corresponds to a possible evolution for the system from site a at time 0 to site c at time t while going through sites $s_1, s_2, \ldots$ at intermediate times $t/M, 2t/M, \ldots$. Now for large M we expand as in Euler's method,

$$\langle c|e^{-iHt}|a\rangle = \sum_{(s_0=a,s_1,s_2,\cdots s_{M-1},s_M=c):\ 1\le s_j\le L}\ \prod_{r=1}^{M}\langle s_r|1-iHt/M|s_{r-1}\rangle + O(1/M)\,, \tag{14}$$

and since H includes only nearest-neighbor hopping, $\langle s_r|1-iHt/M|s_{r-1}\rangle$ vanishes for $|s_r - s_{r-1}| > 1$. Thus, the only sequences $(s_0, s_1, s_2, \cdots s_{M-1}, s_M)$ that contribute to the sum in the large-M limit are those where $|s_{r+1} - s_r| \le 1$.

Consider one such sequence $P{=}(s_0{=}a, s_1, s_2, \cdots, s_{M-1}, s_M{=}c)$ contributing to the sum in Supplementary Equation (14). There will be some time step R at which the sequence P first enters region B (and thus s_R is the leftmost site in region B while s_{R-1} is the rightmost site in region A). Similarly, let R' be the last time step at which the sequence P is in region B (so $s_{R'}$ is the rightmost site in region B while $s_{R'+1}$ is the leftmost site in region C). We may write out the sequence P in more detail as $P = (s_0, s_1, \cdots, s_{R-2}, s_{R-1}, s_R, s_{R+1}, \cdots s_{R'-2}, s_{R'-1}, s_{R'}, s_{R'+1}, s_{R'+2}, \cdots, s_{M-1}, s_M)$.

Now we construct a twin sequence $\tilde{P}$ that starts from site $L+1-a$ in region C and ends at site $L+1-c$ in region A: $\tilde{P} = (L+1-s_0, L+1-s_1, \cdots, L+1-s_{R-2}, L+1-s_{R-1}, s_{R'}, s_{R'-1}, s_{R'-2}, \cdots, s_{R+1}, s_R, L+1-s_{R'+1}, L+1-s_{R'+2}, \cdots, L+1-s_{M-1}, L+1-s_M)$. The first portion of sequence $\tilde{P}$ is the mirror image (in region C) of the first portion of sequence P (in region A), the middle portion of sequence $\tilde{P}$ (which includes all visits to region B) is just the middle portion of sequence P traversed in reverse order, and the last portion of sequence $\tilde{P}$ (in region A) is the mirror image of the last portion of sequence P (in region C). Now sequences P and $\tilde{P}$ visit the same sites in region B (in reverse order), and H is real symmetric, so reversing the order in which sites are visited does not change the amplitude, $\langle s_r|1-iHt/M|s_{r-1}\rangle = \langle s_{r-1}|1-iHt/M|s_r\rangle$. Thus, the amplitude associated with sequence $\tilde{P}$ is identical to the amplitude associated with sequence P. Since each sequence P contributing to $\langle c|e^{-iHt}|a\rangle$ in Supplementary Equation (14) has a twin $\tilde{P}$ contributing to $\langle L+1-c|e^{-iHt}|L+1-a\rangle$, taking $M\to\infty$ we have

$$\langle c|e^{-iHt}|a\rangle = \langle L+1-c|e^{-iHt}|L+1-a\rangle\,. \tag{15}$$

Now we may generalize Supplementary Equation (15) to a superposition initial state $|\Psi(0)\rangle = \sum_{a=1}^{L_A}\kappa_a|a\rangle$ spread over several sites in region A. By linearity,

$$\langle c|e^{-iHt}\,|\Psi(0)\rangle = \langle L+1-c|e^{-iHt}\left|\tilde{\Psi}(0)\right\rangle\,, \tag{16}$$

where $\left|\tilde{\Psi}(0)\right\rangle = \sum_{a=1}^{L_A}\kappa_a|L+1-a\rangle$ is the mirror image under parity of state $|\Psi(0)\rangle$.

Finally, we may sum over the final state,

$$\sum_{c\in C}\left|\langle c|e^{-iHt}\,|\Psi(0)\rangle\right|^2 = \sum_{c'\in A}\left|\langle c'|e^{-iHt}\left|\tilde{\Psi}(0)\right\rangle\right|^2\,, \tag{17}$$

proving the theorem.

□

Supplementary Note 3: TIME NEEDED TO TRAP A PARTICLE

Let us consider the regime of the underbarrier resonant trapping ($U = h/2$) with the initial state

$$|\Psi(0)\rangle = \hat{c}^\dagger_{1,\downarrow}\hat{c}^\dagger_{1,\uparrow}|0\rangle, \tag{18}$$

i.e., a doublon positioned at the leftmost site of the lattice of any size. We define j^* as a site where the height of the barrier satisfies $V^{\rm ex}_{j^*} = h/2$. Supplementary Figures 3 (a) and (b) show the time evolution of $\langle\hat{n}_{h/2}\rangle$. As the system size increases, the particles have more options to distribute before reaching the barrier, resulting in lower values of $\langle\hat{n}_{h/2}\rangle$. Additionaly, for larger sys-

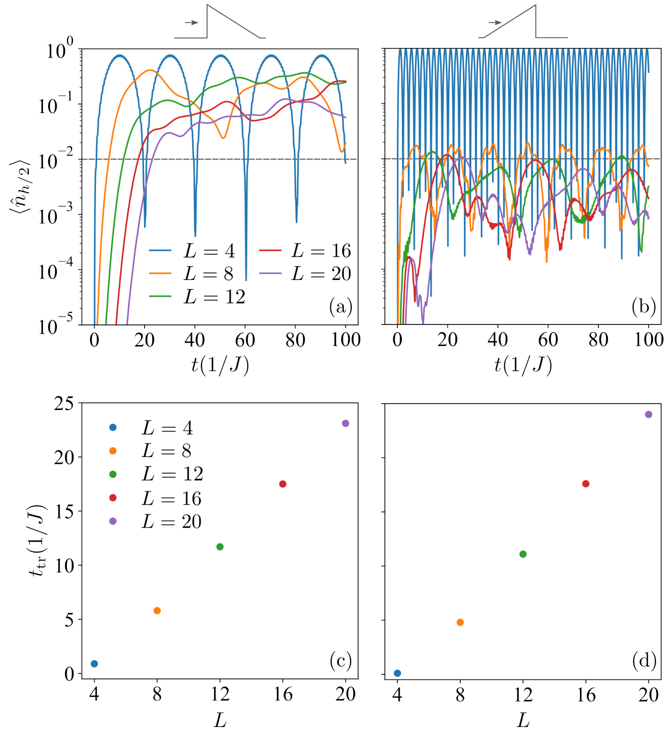

Supplementary Figure 3. **Scaling of the time to reach site j^* within underbarrier trapping regime.** (a) and (b): Time dependence of $\langle \hat{n}_{h/2} \rangle$ for different system sizes. Dashed gray lines correspond to $\langle \hat{n}_{h/2} \rangle = 0.01$. (c) and (d): Time $t_{\rm tr}$ needed to reach site j^* as a function of L, i.e., moment of time when $\langle \hat{n}_{h/2} \rangle$ exceeds 0.01. The right (left) column corresponds to the vertical (angled) orientation of the barrier. For numerical simulations, we used the following parameters: $U = 10J$, $h = 20J$. The initial configuration contains a doublon at the leftmost site of the system.

tem sizes, it takes longer for a particle to reach the site j^*. Supplementary Figures 3 (c) and (d) illustrate how the time $t_{\rm tr}$ required to trap a particle at j^* scales with the system size L, where $t_{\rm tr}$ is identified as the moment when $\langle \hat{n}_{h/2} \rangle$ first exceeds 0.01. As can be seen, it displays nearly linear dependence on L, except for $L = 4$, where the deviation is attributed to finite-size effects.

SUPPLEMENTARY REFERENCES

[1] L. M. Falicov and J. C. Kimball, Simple model for semiconductor-metal transitions: SmB_6 and transition-metal oxides, Phys. Rev. Lett. **22**, 997 (1969).
[2] J. K. Freericks and V. Zlatić, Exact dynamical mean-field theory of the Falicov-Kimball model, Rev. Mod. Phys. **75**, 1333 (2003).
[3] R. Lemański, K. J. Kapcia, and S. Robaszkiewicz, Extended Falicov-Kimball model: Exact solution for the ground state, Phys. Rev. B **96**, 205102 (2017).
[4] K. J. Kapcia, R. Lemański, and S. Robaszkiewicz, Extended Falicov-Kimball model: Exact solution for finite temperatures, Phys. Rev. B **99**, 245143 (2019).
[5] J. J. Sakurai and J. Napolitano, *Modern Quantum Mechanics (3rd Edition)* (Cambridge University Press, 2020).
